# Self-Assembled Monolayers Coated Porous SnO_2_ Film Gas Sensor with Reduced Humidity Influence

**DOI:** 10.3390/s21020610

**Published:** 2021-01-17

**Authors:** Cheonji Lee, Sunjong Oh, Seung-Chul Park, Ho-Nyun Lee, Hyun-Jong Kim, Jinkee Lee, Hyuneui Lim

**Affiliations:** 1 Department of Nature-Inspired Nanoconvergence Systems, Korea Institute of Machinery and Materials (KIMM), 156 Gajeongbuk-Ro, Yuseong-Gu, Daejeon 34103, Korea; cjlee11@kimm.re.kr (C.L.); ssun@kimm.re.kr (S.O.); scpark@kimm.re.kr (S.-C.P.); 2 School of Mechanical Engineering, Sungkyunkwan University, 2066 Seobu-Ro Jangan-Gu, Suwon 16419, Korea; lee.jinkee@skku.edu; 3 Surface Technology R&D Group, Korea Institute of Industrial Technology (KITECH), 156 Gaetbeol-ro, Incheon 21999, Korea; hnlee@kitech.re.kr (H.-N.L.); hjkim23@kitech.re.kr (H.-J.K.)

**Keywords:** self-assembled monolayers, porous SnO_2_ film, CO sensing, humidity, superhydrophobicity, water vapor repellency

## Abstract

Metal-oxide sensors, detect gas through the reaction of surface oxygen molecules with target gases, are promising for the detection of toxic pollutant gases, combustible gases, and organic vapors; however, their sensitivity, selectivity, and long-term stability limit practical applications. Porous structure for increasing surface area, adding catalyst, and altering the operation temperature are proposed for enhancing the sensitivity and selectivity. Although humidity can significantly affect the property and stability of the sensors, studies focusing on the long-term stability of gas sensors are scarce. To reduce the effects of humidity, 1H, 1H, 2H, 2H–perfluorooctyltriethoxysilane (PFOTS) was coated on a porous SnO_2_ film. The interconnected SnO_2_ nanowires improved the high surface area, and the PFOTS coating provided superhydrophobicity at water contact angle of 159°and perfect water vapor repellency inside E-SEM. The superhydrophobic porous morphology was maintained under relative humidity of 99% and operating temperature of 300 °C. The CO gas sensing of 5, 20, and 50 ppm were obtained with linearity at various humidity. Flame detection was also achieved with practical high humidity conditions. These results suggest the simple way for reliable sensing of nanostructured metal-oxide gas sensors with high sensitivity and long-term stability even in highly humid environments.

## 1. Introduction

Semiconducting metal-oxide gas sensors have been studied for their significant applications in security, industrial safety, automobiles, medical diagnosis, and environmental monitoring [1,2,3]. Among several metal-oxide gas sensors, tin (IV) oxide (SnO_2_), which is an important n-type oxide and wide band gap (3.6 eV) semiconductor, is predominantly used as an active layer for gas sensors owing to its high sensitivity, rapid response, recovery property, cheap manufacturing cost, and low operating temperatures, among others [4]. The detection mechanism of SnO_2_-based gas sensors can be explained by the changes in the resistance of the semiconducting SnO_2_ layer caused by the adsorption of oxygen or reaction with target gas molecules [5,6]. Recently, various nanostructures for SnO_2_ gas sensing layers have been developed and tested to improve the gas sensing performance. Nanostructures can provide high sensitivity and rapid response owing to their high surface area and rapid gas diffusion [7,8,9,10]. To develop appropriate nanostructures for gas sensors, several dry process methods have been reported, including sputtering, chemical svapor deposition, pulsed laser deposition, and thermal evaporation [11,12,13,14,15,16,17]. Several structures, such as nanodots, nanobelts, nanohairs, nanowires, nanotubes, and nanoribbons, are prepared and applied to gas sensing with enhanced high sensitivity and low detection limit [18,19,20,21,22,23]. However, it is still difficult to obtain a high surface area and reliable sensor performance for practical applications.

There are diverse parameters that influence gas sensing performance, i.e., affecting the surface reactions, such as property of sensing layer; chemical components, surface electronic state, and nano/microstructures as well as environment condition; temperature and humidity [24,25,26,27]. Especially in nanostructure based gas sensors, environmental humidity is a crucial factor for accurate and reliable gas sensing. Water molecules in humid environments easily attach to the sensor surface and hinder the reaction between the target gas and reaction sites on SnO_2_, thereby inducing a false signal and reducing the sensitivity and long-term stability [1,2,28,29,30]. Furthermore, when the nanostructured SnO_2_ layer is formed on the sensor surface, the water molecules in humid air condense into water droplets on the surface and thereafter infiltrate the nanostructures, causing the collapse of the morphology. This deformation declines the repetitive operation of the gas sensor, resulting in signals with reduced reliability and accuracy. The optimal method for decreasing the humidity effect is to prevent water molecule absorption and water droplet penetration into the gas sensor surface structures. Therefore, the selective control of reactive materials, such as target gas and water molecules, is a major problem in nanostructured metal-oxide gas sensors.

Several studies have introduced self-assembled monolayers (SAMs) coating to increase the selectivity of gas sensors with specific interactions with target materials. The SnO_2_ film sensor utilizes a 3-aminopropyltriethoxysilane as an intermediate medium of the surface to obtain sensitive and selective gas detection operated at some ambient temperature [30]. SAMs also act as block layers on the surface to prevent additional interaction between the sensor surface and unwanted materials except targets [31,32,33]. In particular, hydrophobic surface coating using SAMs shows significant improvements in reducing the undesirable interaction of water molecules owing to the intrinsic coating layer property of low surface energy [33,34,35].

Herein, we propose the simple ways to obtain reliable sensing properties regardless of humidity with a porous SnO_2_ film gas sensor using by vapor SAM coating. The porous film provides numerous reaction sites based on interconnected network nanostructures and the 1H, 1H, 2H, 2H–perfluorooctyltriethoxysilane (PFOTS) SAM coating passivates the surface defects to protect the additional interaction of water molecules. The preparation method and characterization of the PFOTS coated porous SnO_2_ film gas sensor are demonstrated in view of the thermal stability, wetting property, chemical analysis, morphology change, and CO detection under various humidity conditions.

## 2. Materials and Methods

### 2.1. Preparation of the Porous SnO_2_

Film The sensor platform was developed for measuring the gas sensor signal. The platform comprised an alumina (Al_2_O_3_) substrate, designed platinum (Pt) electrodes, and a heater on the back of the substrate (see Figure S4, Supporting Information). The nanostructure was prepared by the thermal evaporation method. A porous SnO_2_ film was deposited on the fabricated alumina surface using a low vacuum thermal evaporator (ULTECH CO., LTD., Deagu, Korea). The source material, SnO_2_ powder (99.99%, TAEWON SCIENTIFIC CO., LTD., Seoul, Korea), was loaded in an alumina-coated tungsten boat placed at the center of a chamber and at 10 cm from the substrate. The substrate was cooled to 10 °C and rotated at 3 rpm for 30 min in SnO_2_ deposition. To obtain a porous morphology with interconnected nanowires, the working pressure was controlled at 0.2 Torr with argon carrier gas [4]. The deposited samples were treated in a furnace at 700 °C for 1 h (see Figure S1, Supporting Information). For the surface analysis of the prepared porous SnO_2_ film, the same deposition process was also performed in silicon (Si) wafers.

### 2.2. SnO_2_ Coating Method of SAMs on Porous SnO_2_ Films

The SAM coating on the porous SnO_2_ film surface was performed by the vapor deposition method. The sample was placed in a plastic container and 100 µL of PFOTS (Sigma-Aldrich CO., LTD, St. Louis, MO, USA) solution was dropped to the bottom of the container. Then, the container was filled with nitrogen gas to prevent the side reaction of PFOTS molecules with moisture. The container was placed in a 100 °C oven for 1 h. After the PFOTS vapor deposition process, the porous SnO_2_ film surface showed superhydrophobicity.

### 2.3. Surface Analysis

To characterize the porous SnO_2_ film surface before and after the PFOTS coating, surface analysis was performed by several methods. X-ray Diffraction (D8 ADVANCE, Bruker, Billerica, MA, USA) is used to confirm the crystal structures before and after annealing with Cu Kα radiation. The surface chemical compositions were investigated using an energy-dispersive X-ray spectroscope (EDAX, AMETEK, Inc., Berwyn, PA, USA) and X-ray photoelectron spectroscope (XPS, PHI 5000 VersaProbe, ULVAC PHI). Surface morphologies were obtained using a field emission scanning electron microscope (FE-SEM, NOVA NanoSEM 200, FEI Co., Hillsboro, OR, USA). Superhydrophobicity of the surfaces was determined by measuring the water contact angles with a contact angle meter (DM-50, Kyowa Interface Science Co., Ltd., Niiza-city, Japan) and by monitoring the condensation behavior inside an environmental scanning electron microscope (E-SEM, Quanta 250, FEI Co., Hillsboro, OR, USA).

### 2.4. Gas Sensing Measurement

For measuring the sensing properties, a custom-built experimental apparatus was used to control the humidity (see Figure S2, Supporting Information). The gas sensor was placed at the center of the chamber and electrically connected to measure the change in resistance with different concentrations (5, 20, and 50 ppm) of carbon monoxide (CO). The relative humidity (RH) in the chamber was adjusted to 20%, 40%, 70%, and even 99% (at 25 °C) to check the effect of humidity on sensing. The operational temperature of the gas sensor was maintained at 300 °C, wherein SnO_2_ is sensitive to CO by heating electrodes from the back of the substrate. Gas sensor parameters, including response and recovery time, were obtained. For reliable measurements, the gas sensor was preconditioned for 1 h at the measurement temperature under flowing dry air before each set of measurements. CO gas was injected for 10 min to stabilize the gas sensor and the response and recovery time were calculated using the time required to achieve 90% of the total resistance change in the case of absorption and desorption, respectively.

## 3. Results and Discussion

**Figure 1:**
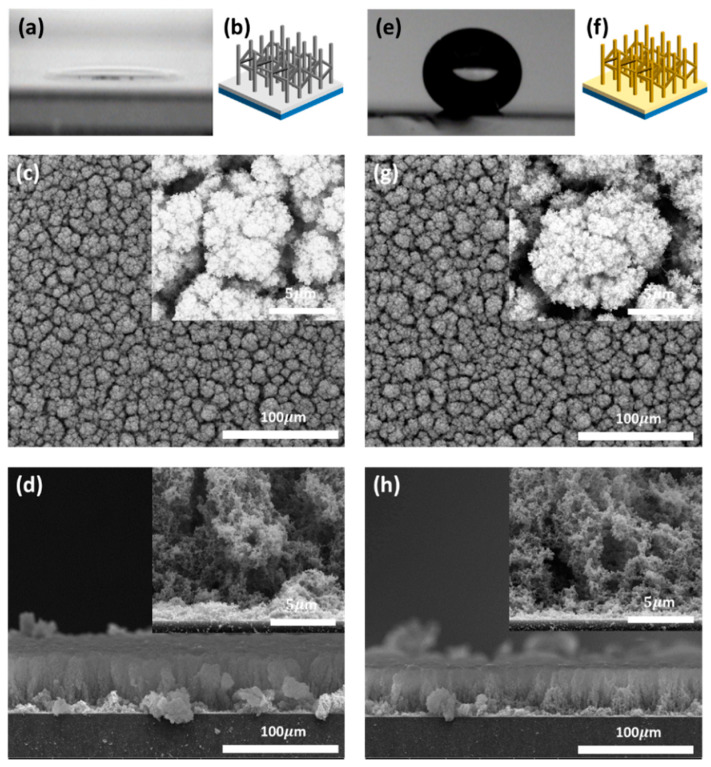
Water contact angle and FE-SEM images of the developed porous SnO_2_ film before and after PFOTS coating. (**a**) Water contact angle of the porous SnO_2_ film. (**b**) Schematic of the grown SnO_2_ film. (**c**) Top view and (**d**) cross-sectional view of the SEM image of the porous SnO_2_ film. (**e**) Water contact angle of the PFOTS coated porous SnO_2_ film. (**f**) Schematic of the grown PFOTS coated SnO_2_ film. (**g**) Top view and (**h**) cross-sectional view of the SEM image of the PFOTS coated porous SnO_2_ film.

Figure 1 shows the water contact angle and surface morphology of the developed porous SnO_2_ film before and after PFOTS coating. A porous SnO_2_ film prepared by thermal evaporation at a relatively low vacuum process (0.2 Torr) is hydrophilic with a water contact angle of 17°, as shown in Figure 1a. The schematic of the grown SnO_2_ film is shown in Figure 1b. The crystal structure was determined by X-ray diffraction (XRD) in Figure S1, Supporting Information. The average crystallite size was estimated as 6.1 nm from tetragonal SnO_2_ (T-SnO_2_) diffraction patterns using Scherrer’s equation. Figure 1c,d show the specific porous morphology with interconnected nanowires of the SnO_2_ film. The top FE-SEM image of the grown SnO_2_ film shows the rainforest-like morphology and the cross-sectional FE-SEM image also shows the lush tree-like porous SnO_2_ film analogous to rainforests. The film was approximately 68 µm thick. The inset images show the porous and interconnected nanostructures. The main factors to determine the sensitivity and detection limit of the gas sensor are closely related to the surface morphology such as porosity, particle size and specific surface area. In particular, the gas sensors composed of small particle sizes shows the improved gas response by increasing the part of the depletion region on the surface. Because the porous SnO_2_ film was composed of nanowires and a large amount of open pore, CO gas easily diffused into the film surface and reacted with the oxygen adsorbates. Therefore, this distinctive SnO_2_ film structure provides excessive reaction sites based on the large surface area and can increase the sensitivity and decrease the detection limit [12]. Despite these advantages, the porous SnO_2_ film can be affected by the humid environment because the reaction sites of SnO_2_ also respond to water molecules. To reduce the influence of humidity on sensing, the prepared porous SnO_2_ film was treated with PFOTS as a hydrophobic coating layer. PFOTS is a self-assembled monolayer molecule that modifies the surface to a low surface energy state [34]. A porous SnO_2_ film consists of numerous pores and thin nanowires, that can easily aggregate by capillary forces if the surface is exposed to moisture or water and then dried in air. Therefore, PFOTS coating was performed using by vapor deposition method to maintain the porous morphology. After the PFOTS coating, the water contact angle becomes 159° exhibiting superhydrophobicity, as shown in Figure 1e. Remarkably, the water droplet was not attached to the surface showing the extreme water repellency due to the specific surface morphology and low surface tension chemical coating. Based on the wetting behavior, it is proposed that the significant affinity of PFOTS with SnO_2_ enables the SAMs on the nanowire branches of the SnO_2_ film without any physical deformation, as shown in Figure 1f. Figure 1g,h show the morphology of the porous SnO_2_ film without changes in the FE-SEM images after PFOTS coating. Therefore, PFOTS coated layers does not affect the morphology because the thickness of PFOTS monolayer is below 2 nm if the monolayer is assembled conformably.

**Figure 2:**
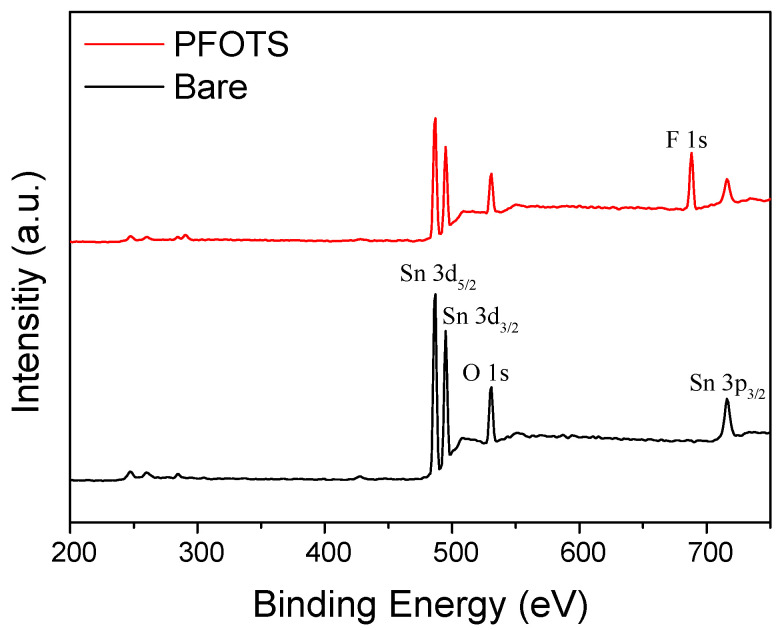
XPS survey spectrum of porous SnO_2_ film before and after PFOTS coating.

Surface analysis of the PFOTS monolayer formation on the porous SnO_2_ film cannot validate a complete coating of the surface owing to the large surface area and randomly interconnected complex morphology. Initially, chemical composition analysis was performed by XPS, as shown in Figure 2. The XPS survey spectrum of the porous SnO_2_ film on the Si wafer shows tin (Sn) and oxygen (O), whereas the XPS spectrum of the PFOTS coated porous SnO_2_ film has a F_1S_ peak at 687.5 eV, indicating the presence of PFOTS coating. Although not shown in the results, the EDAX analysis also showed that F is distributed evenly on the PFOTS coated porous SnO_2_ films using a mapping method.

**Figure 3:**
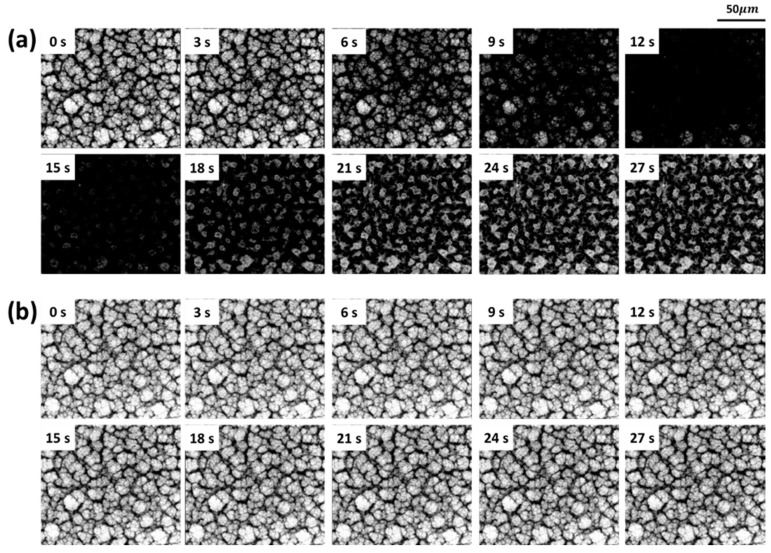
Top view of the water condensation process in E-SEM. (**a**) Case of bare porous SnO_2_ and (**b**) case of PFOTS coated porous SnO_2_. Temperature of the sample holder is maintained at 4 °C. Pressure is regulated with water vapor from 400 Pa to 800 Pa in the E-SEM.

The quality of the PFOTS coating was investigated in a condensation experiment using an E-SEM. To obtain resistance in humid conditions, the surface is completely covered with PFOTS molecules. A porous SnO_2_ film and a PFOTS coated porous SnO_2_ film on a Si wafer were placed in an E-SEM sample holder and the temperature of the sample holder was maintained at 4 °C. The pressure in the E-SEM was regulated with water vapor from 400 to 800 Pa to observe a condensation behavior. Figure 3a shows the condensation process of bare porous SnO_2_ film over time. The brightness of the screen reduces with increasing pressure because of the condensed water on the porous SnO_2_ film. The film-wise condensation occurred rapidly, 3 s, and formed a complete water layer at 12 s. As the pressure decreases, the brightness of the screen is recovered, showing the surface morphology. Moreover, the morphology of the porous SnO_2_ film changes by aggregating each nanostructure. In contrast, for a PFOTS-coated porous SnO_2_ film, although the pressure in the E-SEM was altered to 800 Pa, there was no change in the morphology, as shown in Figure 3b. This behavior shows that water vapor does not interact with the PFOTS coated porous SnO_2_ film and the perfect water vapor repellency operates even at dew point temperature. Therefore, it was confirmed that the PFOTS coated porous SnO_2_ film exhibited superhydrophobicity at nanoscale and the porous SnO_2_ film was coated by PFOTS with almost complete coverage.

**Figure 4:**
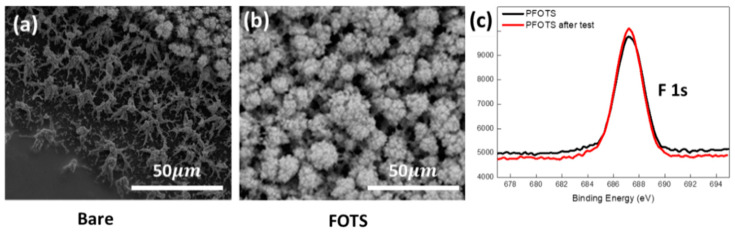
Top view of (**a**) bare and (**b**) PFOTS coated SnO_2_ film after heat treatment at 300 °C, 99% RH for 1 h, and (**c**) XPS spectrum of F_1S_ peak of PFOTS coated SnO_2_ film before and after heat treatment.

The desorption of oxygen adsorbates, the reactivity between the adsorbates and CO gas, and the penetration depth of CO gas into the SnO_2_ film increased as the operation temperature increased [10]. For this reason, the operating temperature is crucial for determining the sensitivity and selectivity of the target gas in a semiconducting metal-oxide gas sensor. From previous studies, nanostructured SnO_2_ based sensors have prominent signals and fast reaction time for CO gas at 300 °C [4,36]. Therefore, the thermal stability of the PFOTS coating at 300 °C with 99% RH in chamber (25 °C) was investigated by the contact angle measurement for superhydrophobicity, FE-SEM analysis for morphology, and XPS analysis for chemical composition. The 300 °C heat treatment for PFOTS coated samples grown on the alumina sensor platform in 99% RH environment was performed for 30 min with the heater at the back of the sensor platform in a custom-built experimental chamber that controls the temperature and humidity (see Figure S2, Supporting Information). After heating, the contact angle of the PFOTS coated sample was maintained at 159 °C and water droplet repellency was retained. The nanostructures on the bare porous SnO_2_ film were aggregated and collapsed by moisture, and the rainforest-like morphology on the PFOTS coated porous SnO_2_ film remained intact, as shown in Figure 4a,b, respectively. The chemical binding state of fluorine (F) originating from PFOTS coating in XPS was also observed to be almost the same for samples before and after heat treatment, as shown in Figure 4c. The F_1S_ peak at 687.5 eV in the XPS analysis shows that there is no decomposition or deformation in the C-F bonds with the unchanged binding energy and intensity. Therefore, the surface characterization results indicate that there were no changes in the surface morphology and chemical composition. The PFOTS coating can be used as a hydrophobic layer at operation temperature and high humidity.

**Figure 5:**
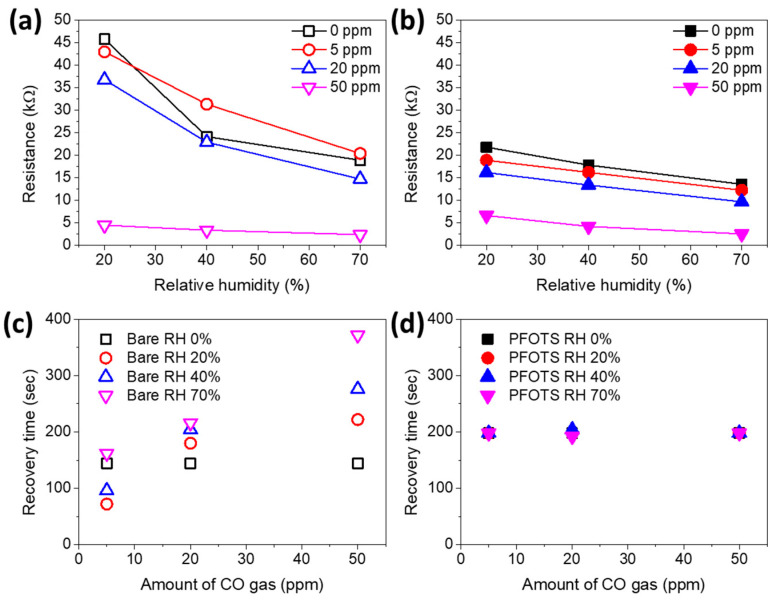
The resistance values (**a**,**b**) and recovery times (**c**,**d**) of both bare and PFOTS coated porous SnO_2_ film gas sensor according to different relative humidity and amount of CO gas.

For a practical application of PFOTS coated porous SnO_2_ film gas sensors, the sensing properties of the porous SnO_2_ film before and after PFOTS coating were examined by CO detection in a custom-built chamber with controlled humidity. The improved sensitivity and lower detection limit of the porous SnO_2_ film were already verified based on the interconnected nanowire morphology of high surface area [10]. The gas response and recovery time of sensing were obtained from real data (see Figure S3, Supporting Information) by measuring the resistance changes with 5, 20, and 50 ppm of CO concentrations according to the humidity at 20%, 40%, and up to 70% with sensor platform temperature at 300 °C, as shown in Figure 5. The gas response of the n-type SnO_2_ sensor contributes to the decreased resistance values with increasing concentrations of CO gas or the presence of water molecules by reducing the SnO_2_ surface. The influence of humidity is explained based on the metal-oxide conduction mechanism, which depends on the adsorption of water molecules by the reaction between the surface oxygen and water molecules, resulting in a decrease in the number of reaction sites on the surface area. In Figure 5a,b, the humidity affects the resistance values for bare and PFOTS coated porous SnO_2_ film gas sensors. This means that false signals are detected in all cases owing to the reaction with water molecules from the humid environment. However, the PFOTS coated sample has lower resistance values compared to the bare sample. It was suggested that the conductivity of n-type semiconductor is increased by fluorine molecules after coating [37,38]. The humidity influence results without CO gas also indicate that PFOTS coated samples is stable and low response values compared to the bare sample (black square symbols in Figure 5a,b). Moreover, the PFOTS coated sample has stability and linearity in resistance changes against humidity and CO gas due to the reduction of dangling bonds and defects. Although if the CO gas is introduced into the chamber, the resistance values decrease depending on the CO concentration in both samples, the response of the PFOTS coated samples was linear depending on the CO concentration. In sensors, linearity is important to determine the exact concentration of target materials. The sensitivity obtained by removing the effect of RH shows the well fitted linear trend line of the PFOTS coated gas sensor with R^2^ as 0.98 or higher (see Figure S4, Supporting Information). Therefore, although the perfect removal of the humidity effect using the hydrophobic PFOTS coating is impossible, passivation and blocking by PFOTS coating help the reliability of the sensing signal. PFOTS coating also provides a stable and reproducible response. Figure 5c,d show the recovery time of each sensing depending on the CO concentration and RH. Whereas the bare sample had different recovery times for each condition and required the prolonged times to recover as the CO concentration and RH increased, the PFOTS coated sample showed almost the same recovery time regardless of the conditions. These results are caused by: the deformation of the nanostructured morphology of the porous SnO_2_ film under humid conditions, as shown in Figure 3a and Figure 4a; and the severe absorption of water molecules limits the desorption of water molecules, causing the deterioration of the sensing property.

**Figure 6:**
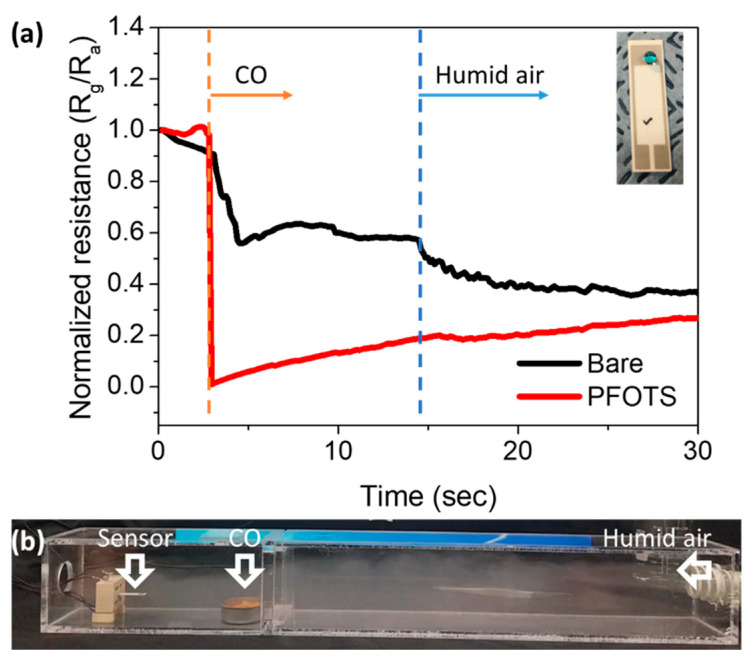
(**a**) The response of bare and PFOTS coated gas sensors in real conditions using a candle flame under humid conditions from a commercial humidifier in an acrylic box and (**b**) the experimental set-up for CO detection from candle flame under humid environment. The resistance of reaction gas is R_g_, and for dry air is R_a_.

The real responses of bare and PFOTS coated gas sensors were observed using a candle flame under humid conditions from a commercial humidifier in an acrylic box, as shown in Figure 6. The bare sample detects CO gas when the candle is turned on and alters the resistance as the humid air is injected strongly because the bare porous SnO_2_ film reacts with both CO gas and humidity. The PFOTS coated sample shows a change in resistance by a candle flame and intact signal from humidity. This indicates that reliable and improved sensing can be achieved with PFOTS coating, and the hydrophobic PFOTS coating is applicable to other ceramic gas sensors to decrease the false signal from water and preserve the nanowire morphology even in humid environments.

## 4. Conclusions

To improve the gas sensing and reduce the influence of humidity, the specific porous morphology of interconnected SnO_2_ nanowires is investigated for high surface area and a PFOTS coating on the surface is performed for water vapor repellency. Vapor deposition of the PFOTS coating on the porous SnO_2_ film maintained the rainforest-like morphology, yielding a water contact angle of 159º, and was stable under 99% RH and 300 °C operating temperature without decomposition and deformation. XPS analysis showed an F_1S_ peak, and the condensation experiment in the E-SEM indicated a superhydrophobic property at the nanoscale. In CO detection at different humidity conditions, the PFOTS coated gas sensor provides a reliable linear response according to the CO concentrations to reduce the influence of humidity significantly. A practical application of detection with candle flame under strong humid conditions showed that the PFOTS coated porous SnO_2_ film gas sensor can be used as a promising sensor without false signals from humid environments. This approach suggests the simple and practical way of nanostructured metal-oxide gas sensors for the reducing humidity influence.

## Supplementary Materials

The following are available online at https://www.mdpi.com/1424-8220/21/2/610/s1, Figure S1: X-ray diffraction (XRD) patterns of porous SnO_2_ films before and after annealing process. Figure S2: Schematic diagram of a custom-built experimental apparatus for controlling humidity. Figure S3: Real time data of bare (**a**) and PFOTS coated porous SnO_2_ film gas sensors (**b**) are obtained at relative humidity 0, 20, 40, and 70 %. Figure S4: Gas sensor sensitivity with removing effect of humidity. (**a**) Bare and (**b**) PFOTS shows sensitivity according to amount of CO; the reaction gas (R_g_) with the humid air signal (R_(a+RH)_). The trend line is drawn with R^2^ at RH 0%. Figure S5: Photos of the gas sensor platform. (**a**) Front image of patterned Pt electrodes and (**b**) back image of heater. (**c**) Schematic image of the porous SnO_2_ gas sensor structure.
