# Supplementary material for: Self-Assembled Monolayers Coated Porous SnO2 Film Gas Sensor with Reduced Humidity Influence
Source: Sensors (Basel). 2021 Jan 17;21(2):610. doi: 10.3390/s21020610 (PMC7829704; doi:10.3390/s21020610)
Supplement: Supplementary file 1 [file sensors-21-00610-s001.pdf]

sensors

MDPI

Communication

Self-Assembled Monolayers Coated Porous SnO_2 Film Gas Sensor with Reduced Humidity Influence

Cheonji Lee [1,2,†], Sunjong Oh [1,†], Seung-Chul Park [1], Ho-Nyun Lee [3], Hyun-Jong Kim [3], Jinkee Lee [2] and Hyuneui Lim [1,*]

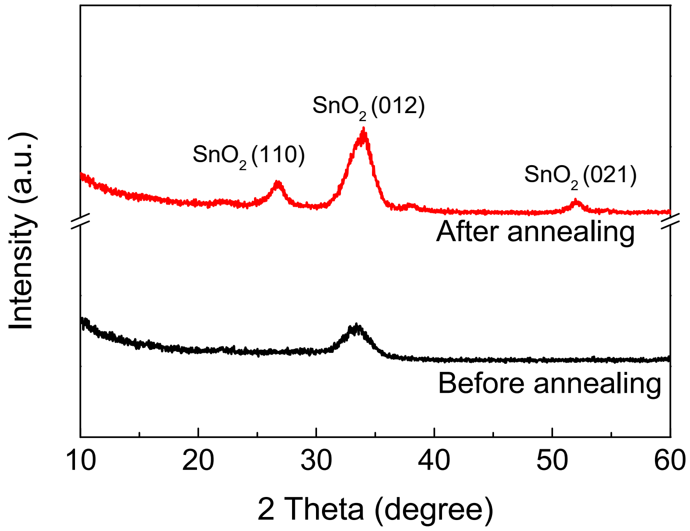

Figure S1. X-ray diffraction (XRD) patterns of porous SnO_2 films before and after annealing process.

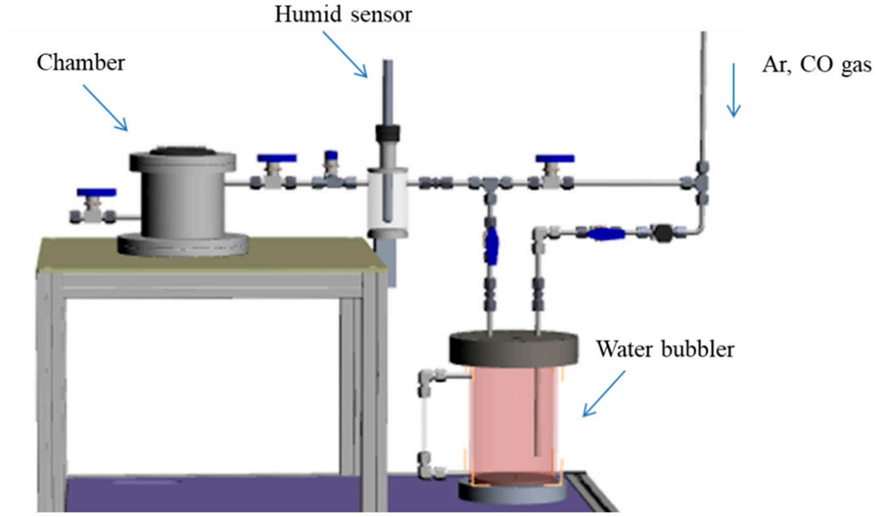

Figure S2. Schematic diagram of a custom-built experimental apparatus for controlling humidity.

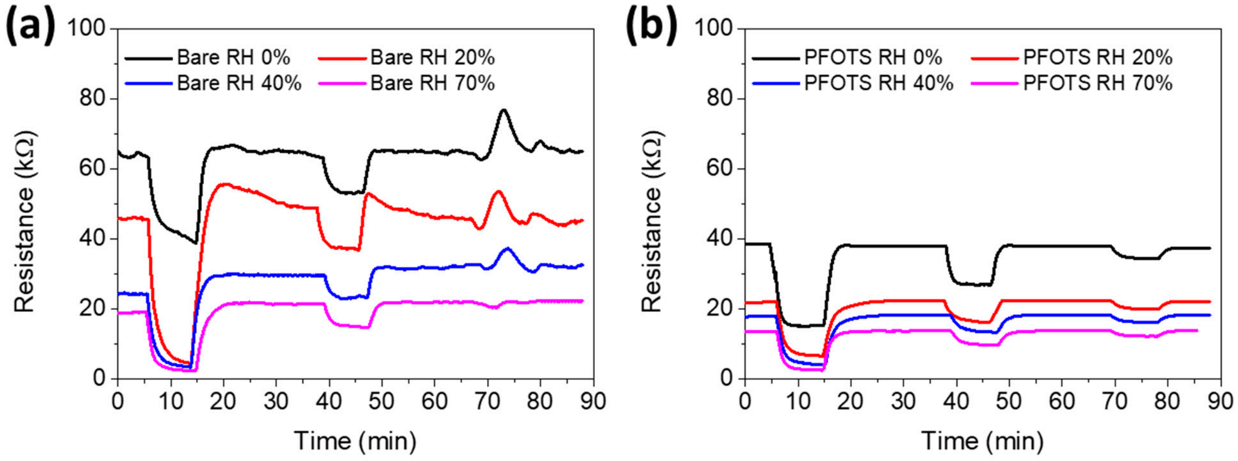

Figure S3. Real time data of bare (a) and PFOTS coated porous SnO_2 film gas sensors (b) are obtained at relative humidity 0, 20, 40, and 70 %.

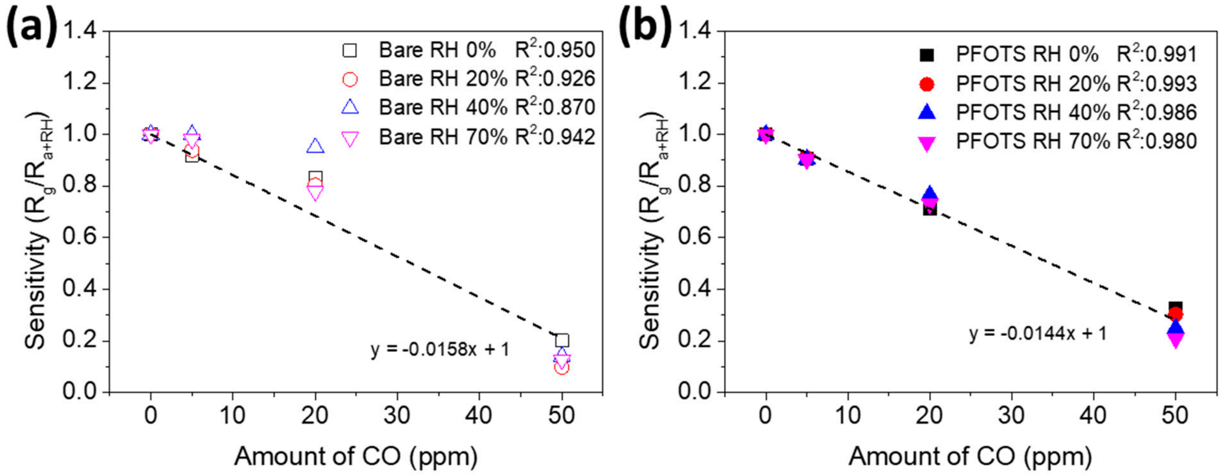

Figure S4. Gas sensor sensitivity with removing effect of humidity. (a) Bare and (b) PFOTS shows sensitivity according to amount of CO; the reaction gas (R_g) with the humid air signal (R_{a+RH}). The trend line is drawn with R^2 at RH 0%.

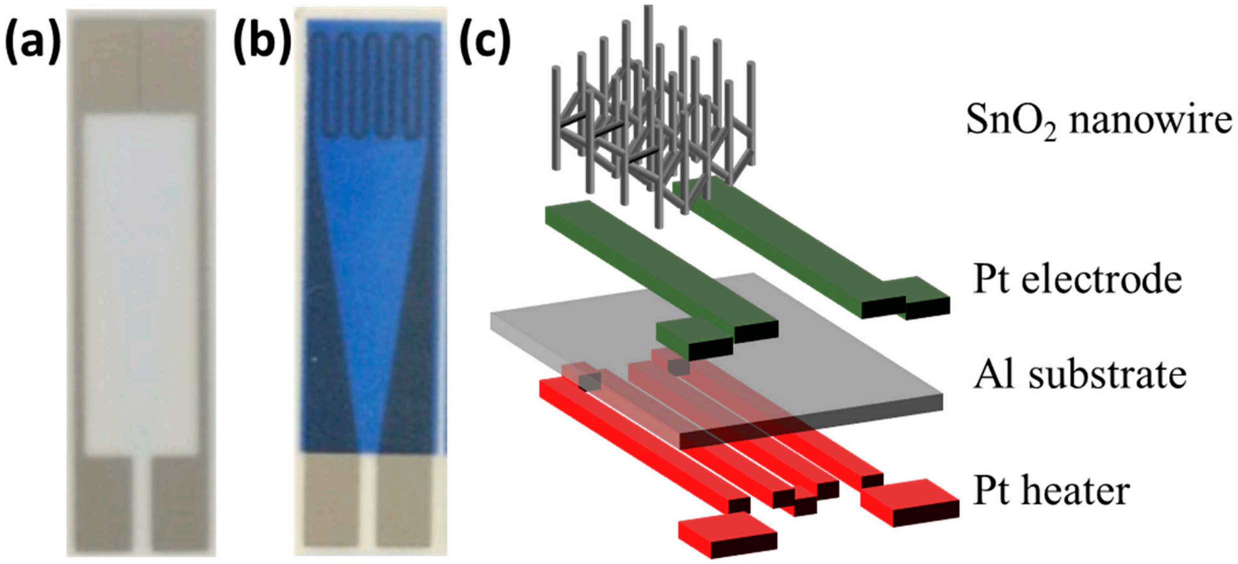

Figure S5. Photos of the gas sensor platform. (a) Front image of patterned Pt electrodes and (b) back image of heater. (c) Schematic image of the porous SnO_2 gas sensor structure.
